# Protective effect of the Japanese traditional medicine juzentaihoto on myelosuppression induced by the anticancer drug TS-1 and identification of a potential biomarker of this effect

**DOI:** 10.1186/1472-6882-12-118

**Published:** 2012-08-09

**Authors:** Kazuo Ogawa, Tatsushi Omatsu, Chinami Matsumoto, Naoko Tsuchiya, Masahiro Yamamoto, Yuji Naito, Toshikazu Yoshikawa

**Affiliations:** 1 TSUMURA Research Laboratories, TSUMURA & CO, 3586 Yoshiwara, Ami-machi, Inashiki-gun, Ibaraki 300-1192, Japan; 2 Department of Molecular Gastroenterology and Hepatology, Kyoto Prefectural University of Medicine, Kajii-cho, Kawaramachi-Hirokoji, Kamigyo-ku, Kyoto, 602-8566, Japan

**Keywords:** TS-1, Bone marrow suppression, Juzentaihoto, SELDI TOF-MS, Albumin fragment

## Abstract

**Background:**

TS-1 is an oral anticancer drug containing a 5-fluorouracil derivative (Tegafur) that is widely used in Japan for the treatment of cancer, especially gastrointestinal tumors. Frequently, however, TS-1 therapy has to be discontinued because of leukopenia. If it were possible to predict the development of bone marrow suppression before the white blood cell (WBC) count had actually decreased, treatment could be improved by strict dosage control and/or the prophylactic administration of hematopoietic drugs. Juzentaihoto (JTT), a traditional Japanese medicine (Kampo), has been reported to activate hematopoiesis and reduce the side effects associated with chemotherapy and radiotherapy. Here, we 1) evaluate the efficacy of JTT in alleviating myelosuppression induced by TS-1 therapy in mice, and 2) explore biomarkers that reflect both induction by TS-1 and alleviation by JTT of bone marrow suppression using a proteomics approach.

**Methods:**

Ten mg/kg of TS-1 was administered to Balb/c mice with or without 1 g/kg of oral JTT for 3, 5 and 7 days. WBC count and ratio of CD34^+^ bone marrow cells (BMCs) were estimated by flow cytometry. Plasma samples were analyzed using surface-enhanced laser desorption/ionization time-of-flight mass spectrometry (SELDI TOF-MS). A biomarker candidate from SELDI profiling was identified using a combination of cation exchange spin column purification, SDS-PAGE, enzymatic digestion and LC-MS/MS.

**Results:**

After administration of TS-1, a significant decrease in WBC count and CD34^+^ BMC ratio were observed at days 5 and 3, respectively. JTT treatment improved WBC count on day 7 and CD34^+^ BMC ratio on days 5 and 7. SELDI analysis highlighted three protein peaks that had increased on day 3 after treatment with TS-1 but remained unchanged in mice co-treated with JTT. One of the three peaks, *m/z* 4223.1, was further investigated and identified as a specific C-terminal fragment of albumin.

**Conclusion:**

This study indicates that bone marrow suppression by treatment with TS-1 in mice might be improved by coadministration of JTT. A C-terminal fragment of albumin was identified as a candidate biomarker for predicting TS-1-induced myelosuppression. However, the sensitivity and specificity of the biomarker candidate must be validated in future clinical studies.

## Background

TS-1 (Taiho Pharmaceutical Co., Ltd, Tokyo, Japan) is an oral fluoropyrimidine derivative consisting of Tegafur (FT) and two modulators, 5-chloro-2,4-dihydroxypyrimidine (gimeracil, CDHP) and potassium oxonate (oteracil potassium, Oxo), an enhancer of FT bioavailability and a soothing agent of gastrointestinal toxicity, respectively [1,2]. TS-1 has been widely used in Japan for the treatment of various tumors due to its high therapeutic efficacy and relative low gastrointestinal toxicity [3]. However, TS-1 does not have a component that is efficient in reducing myelosuppression. Indeed, in clinical practice TS-1 therapy often has to be discontinued because of leukopenia. However, the survival and quality of life of patients may be seriously threatened by the discontinuation of TS-1 therapy.

Currently, a number of traditional Japanese (“Kampo”) medicines are manufactured on a modern industrial scale under strict scientific quality controls. More than 100 Kampo medicines have been approved as ethical drugs by the Ministry of Health, Welfare and Labor of Japan, and are used clinically for the treatment of a wide variety of diseases. One such Kampo medicine, Juzentaihoto (JTT), is known to improve the general systemic condition of cancer patients by reducing the adverse effects of chemotherapy, radiation therapy and surgical treatment [4]. Experimental studies suggest that JTT increases the number of hematopoietic stem cells [5] and reduces *cis*-diamminedichloroplatinum(II) (CDDP)-induced severe bone marrow toxicity in mice [6]. If JTT can alleviate the bone marrow suppression induced by TS-1, more patients could receive the benefits of this drug for a longer period of time.

A lot of research effort is being devoted to identifying biomarkers that lead to the early diagnosis of various diseases using proteomics technologies. Early and accurate diagnosis of a disease is critically important because the patient can then receive prompt and effective therapy. Often, an otherwise treatable condition becomes untreatable at an advanced stage of the disease. A similar situation can exist during the development of complications brought about by chemotherapy and radiation therapy. Thus, a biomarker that is able to detect the early onset of a side effect, such as bone marrow suppression before a detectable decrease in WBC count, would allow the patient to receive more appropriate treatment (e.g., strict anticancer dosage control and/or prophylactic administration of hematopoietic drugs). Mass spectrometry (MS)-based protein analysis, such as surface-enhanced laser desorption/ionization (SELDI) technology, is a high-throughput proteomic technique that has been used for the discovery of disease-related biomarkers in biological fluids such as plasma, serum, tissue and urine. This technique has been employed to identify a number of biomarkers associated with cancer [7-10], Alzheimer’s disease [11] and non-alcoholic fatty liver disease [12].

In this study, we had two main aims: (i) to assess the efficacy of JTT in alleviating bone marrow suppression induced by TS-1 therapy *in vivo*; and (ii) to identify candidate biomarkers to predict bone marrow suppression by TS-1 treatment before the onset of recognizable leucopenia. To generate a shortlist of candidate peaks, those that were altered by TS-1 treatment but whose change was reversed by JTT-co-treatment were identified. This meant that the peaks might also be used as predictors of whether JTT is effective in preventing or retarding TS-1-mediated bone marrow suppression in the early period of TS-1 treatment.

## Methods

### Drugs

A bulk powder of TS-1 was kindly provided by Taiho Pharmaceutical Co., Ltd, (Tokyo, Japan). TS-1 is an oral fluoropyrimidine derivative consisting of FT and two modulators, CDHP and Oxo, in a molar ratio of 1:0.4:1 [1,2]. FT is a prodrug of 5-fluorouracil (5-FU), and CDHP is a reversible competitive inhibitor of an enzyme involved in the degradation of 5-FU. Therefore, CDHP increases the residence time of the FT-derivative of 5-FU in the tumor tissue, enhancing the antitumor effect. Oxo is mainly distributed in the gastrointestinal tract after *per os* administration to patients, leading to relief of gastrointestinal toxicity induced by 5-FU [13,14].

Juzentaihoto (JTT) was obtained from Tsumura & Co. (Tokyo, Japan). JTT was prepared as a spray-dried powder of a hot water extract obtained from ten medical plants in the following ratio: Astragali Radix (3.0 g), Cinamomi Cortex (3.0 g), Rehmanniae Radix (3.0 g), Paeoniae Radix (3.0 g), Cnidii Rhizoma (3.0 g), Angelicae Radix (3.0 g), Ginseng Radix (3.0 g), Hoelen (3.0 g), Glycyrrhizae Radix (1.5 g) and Atractylodis Lanceae Rhizoma (3.0 g). TS-1 was dissolved in a 0.5% (w/v) hydroxypropylmethylcellulose (HPMC) solution, and JTT was dissolved in distilled water (DW) immediately before use.

### Mice

Six-week-old female specific pathogen-free (SPF) Balb/c mice were purchased from Japan SLC, Inc. (Shizuoka, Japan) and maintained under a constant temperature, humidity and light-controlled environment with free access to food and water. The mice were examined after one-week standardizing diet prior to dosing.

### Treatment of animals and evaluation of myelosuppression

**Figure 1:**
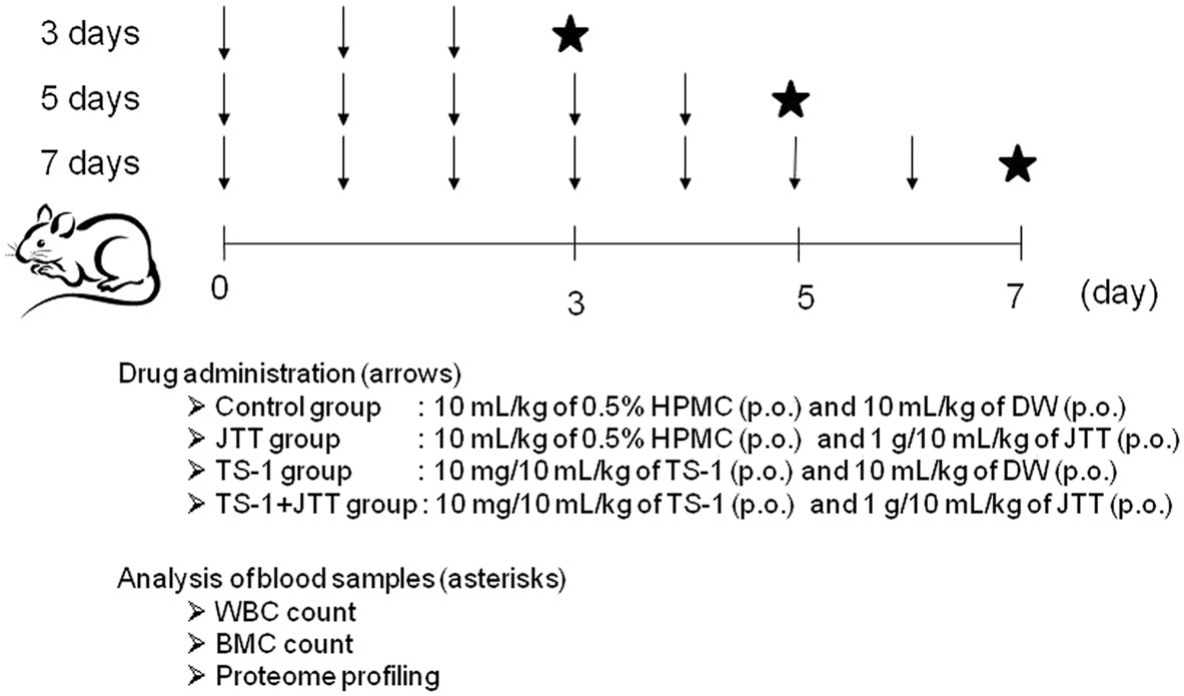
**Outline scheme of the treatment and sampling procedure.** The drugs were orally administered to mice daily for 3, 5 and 7 days. The timing of drug administration is indicated by arrows. Analysis of the blood samples on days 3, 5 and 7 is indicated by asterisks.

Mice were treated orally as follows: 10 mL/kg of 0.5% HPMC and 10 mL/kg of DW were administered to the control group; 10 mL/kg of 0.5% HPMC and 1 g/10 mL/kg of JTT were administered to the JTT group; 10 mg/10 mL/kg of TS-1 and 10 mL/kg of DW were administered to the TS-1 group; and 10 mg/10 mL/kg of TS-1 and 1 g/10 mL/kg of JTT were administered to the TS-1 + JTT group for 3, 5 and 7 days. Mice were anesthetized with diethyl ether and heparinized blood samples were collected from the inferior vena cava of the mice on days 3, 5 and 7 (Figure1). For blood cell analysis, EDTA was added to the blood sample (150 μL at a final concentration of 1 mg/mL) and shaken well. The number of white blood cells (WBCs) was immediately counted using a Sysmex XT-2000i V (Bio-Rad Laboratories Inc., Hercules, CA, USA). The remaining heparinized blood was centrifuged at 880 *g*, 4°C for 10 min and plasma samples were stored at −80°C until SELDI analysis. Bone marrow cells (BMCs) were estimated by flow cytometry analysis. Cell-surface expression of CD34 in BMCs was estimated using a FACS Calibur (Becton, Dickinson and Company, Franklin Lakes, NJ, USA). BMCs were depleted of erythrocytes by addition of lysing buffer (BD Biosciences, San Jose, CA, USA) and then incubated with anti-CD34 antibody (BD Biosciences) conjugated with fluorescein isothiocyanate (FITC) for 30 min on ice. After removal of free antibody, cells were analyzed using a flow cytometer and Cell Quest software (BD Biosciences). The fluorescence intensity of cells gated in high forward and side scatter was quantified. Data are expressed as a percentage of the geometric means of the control mice given 0.5% HPMC and DW.

All procedures were prospectively approved by the Animal Care and Use Committee of Kyoto Prefectural University of Medicine or by the Laboratory Animal Committee of Tsumura & Co.

### SELDI protein profiling

Each plasma sample was analyzed on two different array surfaces: anion exchange (Q10) and cation exchange (CM10). Sinapinic acid (SPA) was used as the energy absorbing molecule (EAM). These materials were purchased from Bio-Rad Laboratories Inc..

Plasma samples were thawed on ice and then mixed at a ratio of 1:9 with urea denaturing buffer (7 M urea, 2 M thiourea, 1% dithiothreitol, 4% CHAPS and 2% ampholyte) or phosphate-buffered saline (PBS) for 10 min on ice. Mixed samples were used for proteomic profiling in duplicates. Binding of proteins and applying of EAM to the array surfaces were performed in a 96-well format bioprocessor using a Biomek2000 automatic system (Beckman Coulter, Inc., Brea, CA, USA) [15,16]. The arrays were analyzed using a Protein Chip System 4000 Reader (PCS4000; Bio-Rad Laboratories, Inc.), which was calibrated for mass accuracy using combined ‘all-in-one’ peptide standards (Bio-Rad Laboratories, Inc.) and cytochrome c (Sigma-Aldrich, St. Louis, MO, USA) for *m/z* 3000–10,000, and protein standard II (Bio-Rad Laboratories, Inc.) for *m/z* 10,000-30,000. Data were averaged from 795 laser shots for each spot. Spectra collection and statistical analyses were performed using the CiphergenExpress (version 3.0.6) software package (Bio-Rad Laboratories, Inc.).

### Purification and identification of biomarker candidates

Ion exchange fractionation was undertaken on a CM Ceramic HyperD F Spin Column (Bio-Rad Laboratories, Inc.) pre-equilibrated with binding/washing buffer (100 mM sodium acetate, pH 4.0). Plasma samples were diluted at a ratio of 1:1.5 in U9 buffer (9 M urea, 2% CHAPS, 50 mM Tris–HCl, pH 9.0) and incubated for 30 min at 4°C on a rotator. Treated samples were then diluted 1:9 in binding/washing buffer and applied to the column, followed by incubation for 120 min at 4°C. Samples applied to the column were first clarified by centrifugation (80 *g*, 4°C for 3 min). The sample applied spin column was centrifuged at 80 g at 4°C for 3 min. The flow-through was collected as Fr.1-1. One hundred and fifty microliters of binding/washing buffer was applied to the column and incubated for 5 min at 4°C on a rotator. The washing step was repeated twice (collected as Fr.1-2 and Fr.1-3). The bound proteins were eluted using 150 μL of elution buffer (50 mM Tris–HCl, pH 8.0) 5 times (Fr.2-1, Fr.2-2, Fr.2-3, Fr.2-4 and Fr.2-5). SELDI TOF-MS was used to monitor each fraction for ions of interest.

Eluted fractions containing biomarker candidates were pooled and concentrated by molecular weight cut-off membrane fractionation (Amicon Ultra-0.5; Millipore Corporation, Billerica, MA, USA). A biomarker candidate was identified using sodium dodecyl sulfate-polyacrylamide gel electrophoresis (SDS-PAGE) coupled with MS analysis. Concentrated fractions were separated by SDS-PAGE using a precast 15% gel and Tris-Tricine buffer. Bands were visualized by silver staining (EzStain Silver; ATTO Corporation, Tokyo, Japan). Gel pieces containing the proteins of interest were excised and subjected to in-gel trypsin digestion, followed by liquid chromatography–tandem mass spectrometry (LC–MS/MS) analysis (performed by APRO Life Science Institute, Inc., Tokushima, Japan). MS/MS spectra were submitted to the database mining tool Mascot (Matrix Science, London, UK) for identification.

### Statistical analysis

For the *in vivo* study, statistical analysis was performed using 1-way ANOVA. Student’s *t*-test was used to determine the significance of differences between the control and TS-1, and between TS-1 and TS-1 + JTT in the dosed testing procedure [17]. In SELDI profiling analysis, statistical analysis was performed using non parametric Kruskal-Wallis *H*-test. Non-parametric Mann–Whitney *U-*test was used to determine the significance of differences between the control and TS-1, and between TS-1 and TS-1 + JTT.

## Results

### Improvement in white blood cell count and bone marrow suppression by JTT

Initially, we examined several dosages of TS-1 and selected a dosage of 10 mg/kg because it induced evident myelosuppression without severe body weight loss. JTT was used at a dosage of 1 g/kg, which has been reported to be effective in various disease models such as mitomycin C- and radiation-induced haematopoietic toxicity [18-20]. Thus, mice were orally given 10 mg/kg of TS-1 followed by 1 g/kg of JTT for 3, 5 and 7 days, and the WBC and BMC counts were determined. The treatment and analysis schedule is described in Figure1.

**Figure 2:**
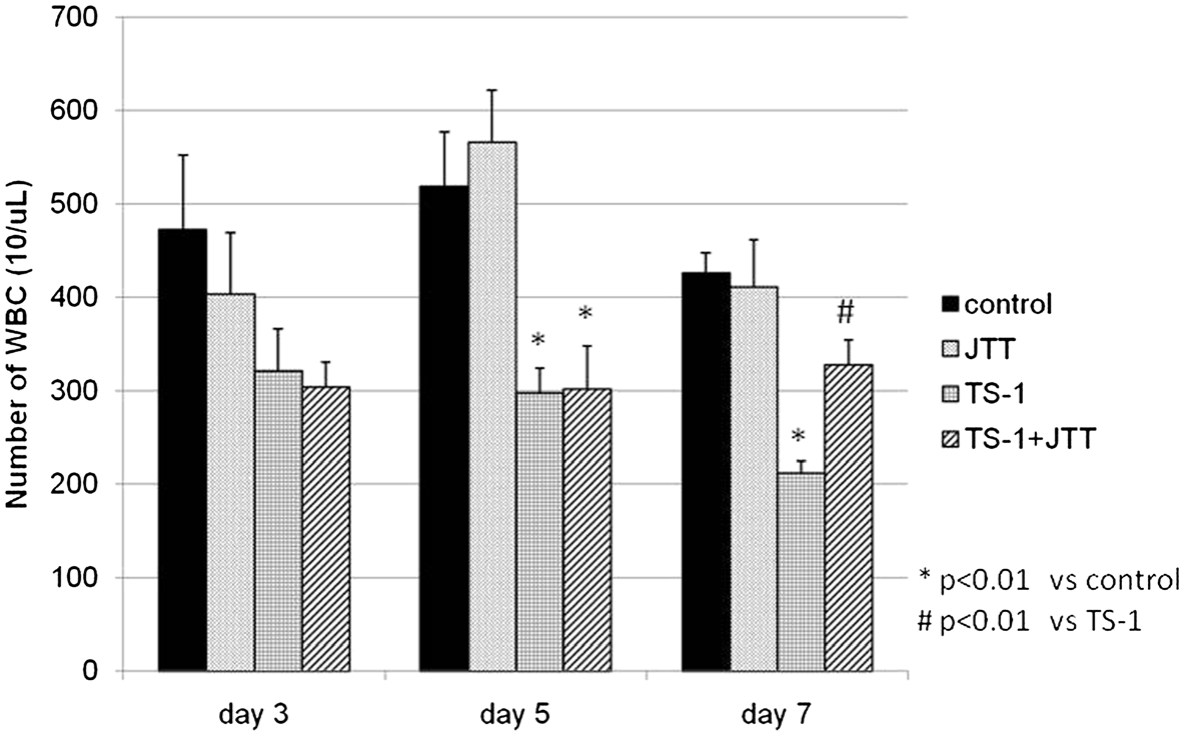
**Comparison of white blood cell count.** Comparison of white blood cell (WBC) count in treated mice on days 3, 5 and 7. Control, treated with 0.5% HPMC and DW; JTT, treated with 0.5% HPMC and 1 g/kg of JTT; TS-1, treated with 10 mg/kg of TS-1 and DW; TS-1 + JTT, treated with 10 mg/kg of TS-1 and 1 g/kg of JTT. Data represent means ± SE.

**Figure 3:**
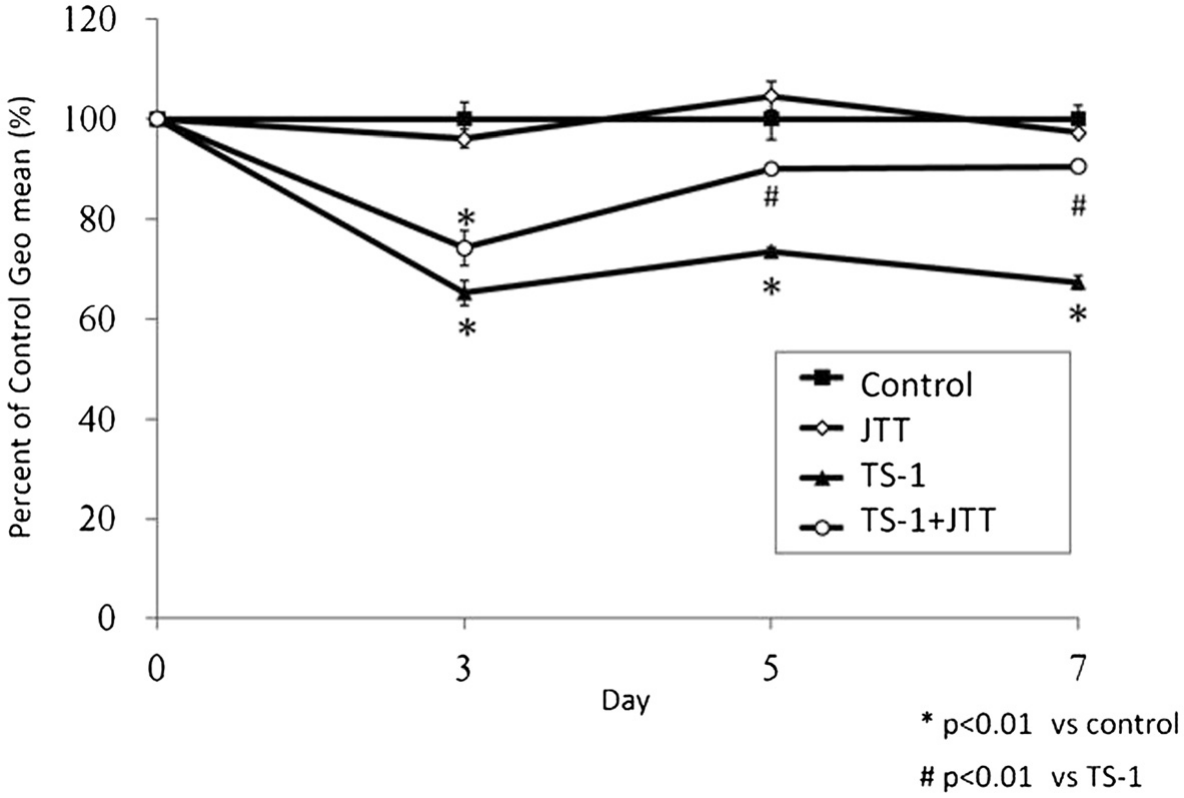
**Comparison of bone marrow cell by geometric mean fluorescence intensity.** BMCs were labeled with anti-CD34 antibody conjugated with FITC. Fluorescent intensities of labeled cells were gated in high forward. Geometric mean of fluorescent intesnsities were calculated and represented as the ratio to the Control values. Data represent means ± SE.

After administration of TS-1, the WBC count was decreased significantly on days 5 and 7, but it was improved by coadministration of JTT on day 7 (Figure2). Bone marrow suppression was evaluated by geometric mean fluorescence intensity (GM) on the *x* axis using a log scale. GM peaks were converted to a linear scale to calculate the percentage of the daily control (%DC). Administration of TS-1 led to a decrease in %DC at 3 days, but %DC was significantly improved by coadministration of JTT at 5 and 7 days (Figure3).

### Investigation of biomarker of myelosuppression by TS-1 by SELDI protein profiling

**Figure 4:**
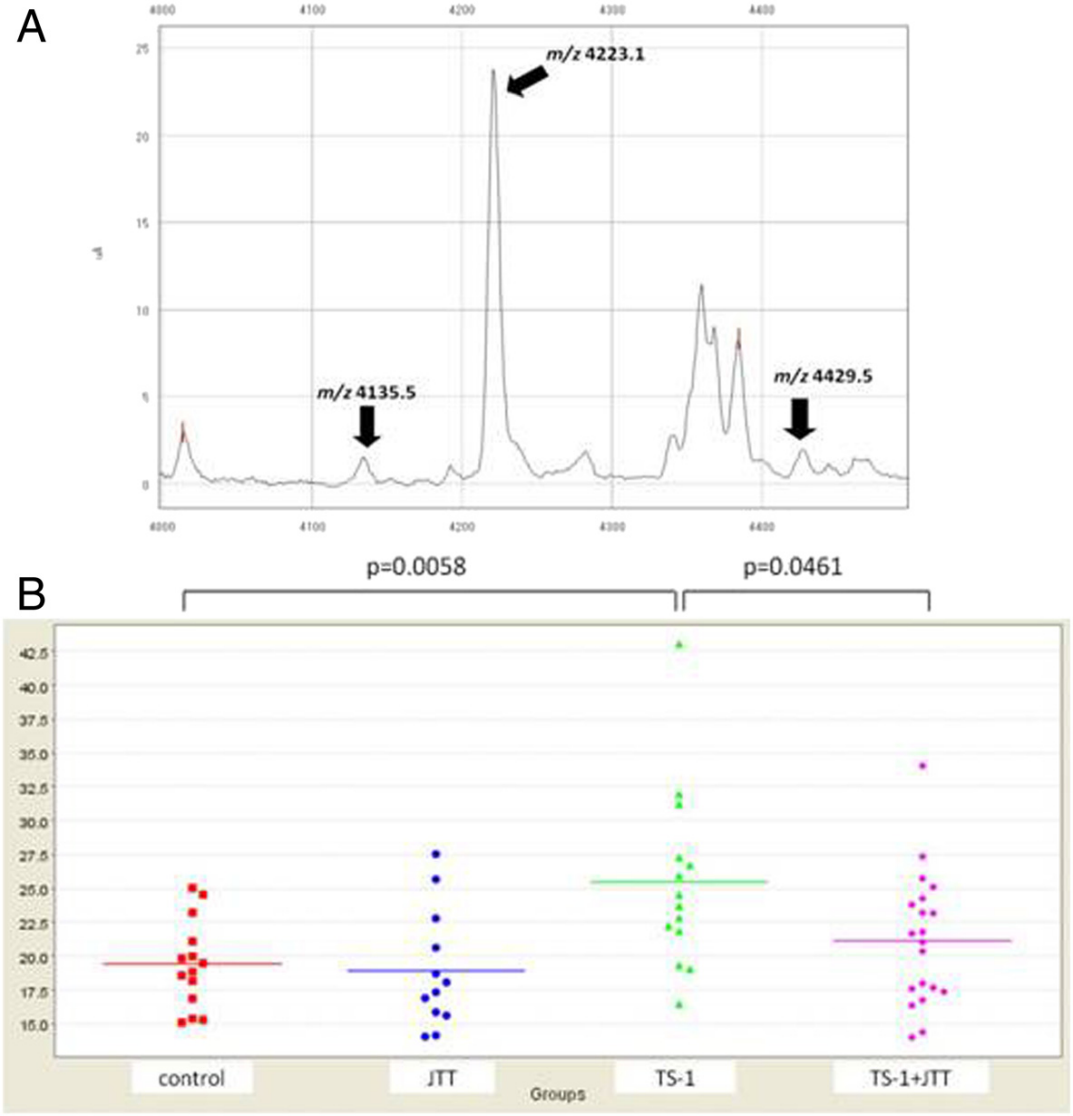
**Detection of biomarker candidates. A**) Spectrum of the peaks of three biomarker candidates (arrows). The protein peaks were detected on a CM10 chip. **B**) Representative SELDI analysis data of the *m/z* 4223.1 peak as a biomarker candidate. In each group, plots indicate the intensity of the peak and bars represent the mean value.

Because chemotherapeutic drugs have a profound effect on blood proteomics, a number of peaks are generally changed with statistical significance in the protein profile. Therefore, it is necessary to include additional criteria for further investigation of possible biomarker candidates. In the present study, because JTT treatment clearly improved TS-1-induced myelosuppression, we searched for peaks that were up- or down-regulated by TS-1 treatment but whose changes were nullified by coadministration of JTT. We compared SELDI profiles among the control group, JTT group, TS-1 group, and TS-1 + JTT group and found that peaks with *m/z* values of 4135.5, 4223.1 and 4429.5 (Figure4A) met the above criterion. The intensity of all three peaks significantly increased on day 3 after administration of TS-1 when the decrease in WBC was still insignificant. In the TS-1 and JTT group, these peaks were not significantly changed as compared with the control group (Figure4B, only *m/z* 4223.1 shown). Intriguingly, on days 5 and 7 there was no difference in the intensity of these three peaks between the control and TS-1 groups (data not shown). As a result, these peaks appeared to be changed prior to a detectable decrease in WBC.

### Identification of biomarker candidates by LC-MS/MS

**Figure 5:**
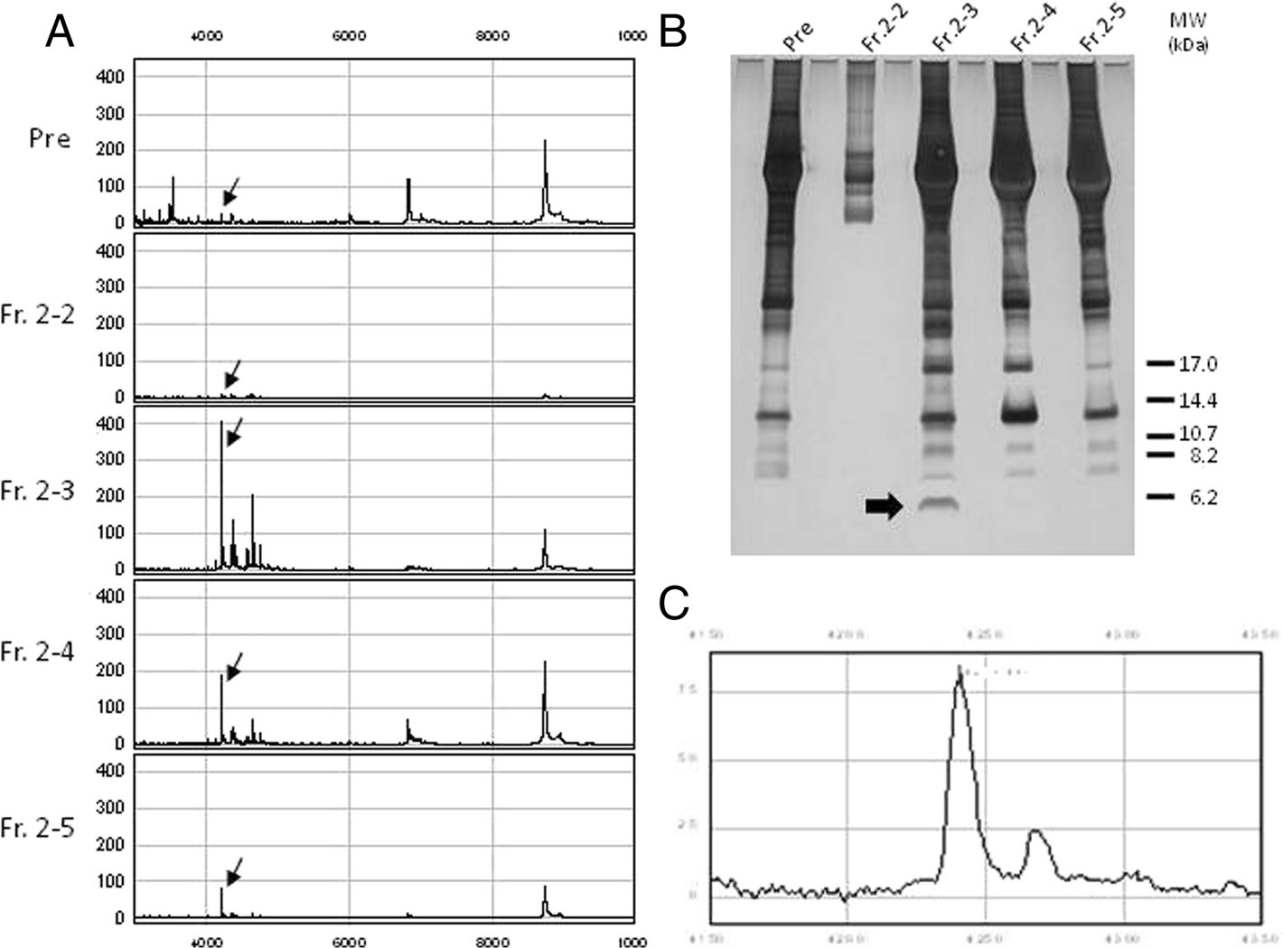
**Purification of a biomarker candidate.** Purification of the biomarker candidate peak at *m/z* 4223.1 involved using a combination of spin column and SDS-PAGE. **A**) Spectra of peak fractions eluted from the cation exchange spin column; before fractionation plasma, Fr. 2–2, Fr. 2–3, Fr. 2–4 and Fr. 2–5. The peaks were detected on a CM10 chip. The candidate peak is marked by an arrow. **B**) Each fraction was separated by SDS-PAGE and protein bands were visualized by silver staining. **C**) The band marked by an arrow in the Fr.2-3 lane was extracted from the gel and then analyzed by SELDI TOF-MS.

The *m/z* 4223.1 peak gave the highest peak intensity among the three biomarker candidates (Figure4A), and was therefore investigated further. First, in order to characterize this candidate marker, fractions eluted from the cation exchange spin column were applied to SELDI protein chip arrays. The candidate marker, *m/z* 4223.1, was detected in the following fractions; Fr.2-2, 2–3, 2–4 and 2–5 (Figure5A). The fractions were concentrated and 1.5 μg (Fr.2-2) and 20 μg (Fr.2-3, 2–4 and 2–5) of protein were separated by SDS-PAGE (Figure5B).

A relatively intense band with a molecular mass of approximately 6500 Da was present in Fr.2-3 but in none of the other lanes. The band was extracted and analyzed by SELDI TOF-MS. The band-extract produced a SELDI peak identical to the protein with *m/z* 4223.1 (Figure5C). The band was subjected to in-gel trypsin digestion followed by LC-MS/MS.

The MS/MS spectra were submitted to Mascot for identification. The mascot research results found two high-scoring peptide sequences that matched the C-terminal region of mouse albumin (Table1A). Thus, the candidate peptide is thought to be a C-terminal fragment of mouse albumin, which contains an additional CKDALA sequence that was not detected in the LC-MS/MS analysis (Table1B). The theoretical mass value of the matched sequence (*m/z* 3644.1) was less than the experimentally determined *m/z* value of 4223.1 using SELDI TOF-MS. However, inclusion of the CKDALA sequence would give a theoretical *m/z* of 4245.8, which is almost identical to *m/z* 4223.1.

**Table 1:** **Peptide sequences of *m/z* 4223.1 determined from the LC-MS/MS analysis**

**A**	**Matched mass**	**Computed mass**	**Residues**	**Sequence**
	1735.8	1735.7	570-584	K.**TVMDDFAQFLDTCCK.**A
	1751.7	1751.7	570-584	K.**TVMDDFAQFLDTCCK**.A Oxidation (M)
	1923.9	1923.9	585-602	K.**AADKDTCFSTEGPNLVTR**.C
	1995.0	1994.9	585-602	K.**AADKDTCFSTEGPNLVTR**.C Propionamide (C)
	1538.7	1538.7	589-602	K.**DTCFSTEGPNLVTR**.C
**B**	551 TALAELVKHK PKATAEQLK**T VMDDFAQFLD TCCKAADKDT CFSTEGPNLV**			
	601 **TR**CKDALA			

A) Peptide sequences determined from LC-MS/MS analysis. B) Amino acid sequence of the C-terminal peptide (from residue 551 to 608) of mouse albumin (Swiss-Prot entry P07724). Matched peptides shown in **Bold**.

## Discussion

5-Fluorouracil (5-FU) and its prodrug TF have been demonstrated to possess potent anti-tumor activity *in vitro*. However, their severe side effects, including myelotoxicity, gastrointestinal toxicity (diarrhea and stomatitis) and central nervous system (CNS) disturbance, mean that it is impossible for these drugs to achieve sufficient clinical benefits. TS-1 was designed with the simultaneous aim of enhancing the efficacy of 5-FU while reducing the associated adverse reaction. Specifically, TS-1 includes both CDHP, an inhibitor of 5-FU degradation, to maintain an effective plasma concentration of FT, and Oxo to reduce the level of gastrointestinal toxicity. By reducing gastrointestinal toxicity, which causes great discomfort to patients, it is possible to use TS-1 for an extended period of time. Thus, TS-1 has become the most frequently-used anti-cancer drug for the treatment of cancer patients in Japan [3].

TS-1 is administered orally for 4 weeks, followed by a 2-week rest. This treatment protocol can be repeated if no serious side effects are apparent. Nonetheless, TS-1 has been reported to cause a variety of adverse reactions, such as anemia, leukopenia, neutropenia, diarrhea, thrombocytopenia, stomatitis, anorexia and proteinuria [21]. In particular, TS-1 suppression of bone marrow is a very serious problem. When a decrease in WBC count is detected in TS-1-treated patients, TS-1 therapy must be immediately discontinued. If the development of TS-1-induced leukopenia can be inhibited, however, the patients can continue to receive therapy. Additionally, the dosage of anticancer agent might even be increased to improve therapeutic gain. This would be a great benefit to patients with gastroenterological cancer.

Our study aimed to solve this problem through three individual steps. First, we investigated whether JTT has a preventive effect on bone marrow suppression induced by TS-1. Second, we attempted to identify candidate biomarkers that can detect myelosuppression before the onset of leukopenia. If we use appropriate precautionary measures such as the strict control of the treatment protocol of TS-1 and/or the prophylactic administration of hematopoietic reagents (e.g. G-CSF), the discontinuation of TS-1 therapy may be avoided or at least delayed. Third, we investigated whether the candidate biomarker could predict whether the patient is a “JTT-responder”.

JTT has been reported to improve the general condition of cancer patients receiving chemotherapy and/or radiation therapy [22]. In mice, it has been suggested that JTT improves the decline in bone marrow function induced by anticancer therapy or radiotherapy and that the effect is mediated, at least partly, by enhancing the proliferation of hematopoietic stem cells. This effect has been demonstrated as an increase in colony-forming units in spleen (CFU-S) and/or granulocyte-macrophage colony-forming cells (CFU-GM) in cisplatin-, mitomycin C-treated or irradiated animals [18,20], and is mediated by mitogenic activity of oleic acid and linolenic acid contained in JTT. The same mechanism may be involved in the inhibition of TS-1-induced leukopenia/bone marrow suppression demonstrated in the present study; however, further extensive studies are necessary to clarify this point.

It has been long supposed that there are distinct groups of responders and non-responders to each Kampo medicine. Certain Kampo medicines have been reported to produce a dramatic therapeutic effect in “responder” patients, but there are always a number of “non-responder” patients. Many clinical trials using Kampo medicines, including more than 10 multicenter, placebo-controlled, double-blind studies, have demonstrated significant beneficial effects of Kampo medicines [23,24]. However, these trials also suggest that it is important to distinguish responders from non-responders at an early stage of therapy to achieve the anticipated therapeutic outcome. Thus, in order to maximize the beneficial effects of Kampo medicines in modern medical practice it is crucially important to identify a suitable biomarker to distinguish a “responder” from a “non-responder”.

Although a huge number of research papers have been devoted to identifying biomarkers, few biomarker(s) have been validated using diagnostic criteria. Currently, proteomic technology is one of the most effective methods for identifying biomarkers. SELDI is an MS-based proteomic technique that has been used in the discovery of disease-related biomarkers derived from biological fluids. We have previously reported several biomarker candidates for Kampo medicines using SELDI [15,16,25]. Among them, haptoglobin alpha 1 chain may be used to predict the efficacy of the Kampo medicine keishibukuryogan in the treatment of rheumatoid arthritis. The strategy used in the present study employed SELDI to find a predictive marker for TS-1-induced leukopenia. Specifically, we focused on protein peaks whose intensity changed significantly prior to a decrease in WBC count. To generate a shortlist of candidate proteins, we focused on peaks that changed upon treatment with TS-1 but were normalized by co-treatment with JTT. Using this approach, we identified three biomarker candidates, which all increased 3 days after administration of TS-1 although coadministration of JTT reduced these changes. Interestingly, the TS-1-induced up-regulation of these biomarker candidates was only transient, because they showed no significant difference on days 5 and 7 as compared with the negative control. Our results suggest that these candidates may be induced in the early phase of TS-1-mediated myelosuppression. As such, these candidate biomarkers may play an important role in the development of a serious adverse effect of TS-1.

We successfully identified one (*m/z* 4223.1) of the three candidate biomarkers as the C-terminal fragment of albumin using a combination of SDS-PAGE and LC-MS/MS analysis. It is implausible that this albumin fragment directly mediates the side effects associated with administration of TS-1, such as leukocytopenia and bone marrow suppression. Therefore, we believe that the albumin fragment may be derived from early molecular events during the onset of bone marrow suppression. Indeed, a decrease in albumin is associated with myelosuppression induced by several chemotherapeutic reagents, including TS-1 [26-30]. Recent studies have suggested that the plasminogen fibrinolytic pathway is required for hematopoietic regeneration [31,32]. Thus, dynamic alteration of the protease-protease inhibitor network might occur in myelosuppression. We intend to investigate whether JTT directly inhibits the protease responsible for producing the albumin fragment of *m/z* 4223.1 in a future study.

Albumin fragments of various length and amino acid sequence have been identified as biomarker candidates for other diseases [33-35]. Those results suggest that the fragments are generated from circulating albumin by specific or nonspecific proteases activated in various disease states. Albumin is the most abundant protein in blood and has a diverse range of functions, including maintenance of intravascular volume and colloid osmotic pressure, binding and transport of various molecules (including hormones, lipid and drugs), antioxidant and anti-inflammatory actions, and exertion of a stabilizing effect on the endothelium [36-39]. Thus, albumin may act as a “buffer” against stress from disease and/or exposure to certain drugs. Various mediators (e.g., inflammatory, oxidative, fibrolytic, chemotactic), including proteases liberated from injured or disabled organs, might be “buffered” or “neutralized” by inactivation upon binding to, reduction with, and digestion of, circulating albumin. It must be noted that fragments of various major serum proteins, such as haptoglobins [15], transferrin [40], fibronectin and apolipoproteins [41], have also been identified as serum biomarkers in various diseases. Abundant serum proteins might represent a reservoir of “scapegoats” for activated proteases. Identification of the protease responsible for generation of the present peptide will contribute to elucidating the mechanisms underlying both the development of bone marrow suppression by TS-1 and the improvement by JTT.

It should be noted, however, that there are species differences in the amino acid sequence between human and murine albumin. The peptide fragment found in this study is supposed to be generated from the cleavage of mouse albumin at lysine 569, which corresponds to lysine 569 of human albumin. The overall identity and similarity of the amino acid sequence of mouse and human albumin (consisting of 609 and 608 AA, respectively) are high (72.3 and 92.9%, respectively), and those of the sequence of the anterior segment of the cleavage sites (541–569) are also high (86.2 and 93.1%, respectively). It therefore is possible that a similar fragment may be generated in human. On the other hand, the identity and similarity of the posterior sequence (570–609) are lower (55 and 82.5%, respectively). Furthermore, recognition of the cleavage site by proteases has varying degrees of stringency depending on each protease. Therefore validation studies in human patients, as well as clarification of the protease responsible for generation of the fragment, are necessary. We plan to execute these studies in the near future in our laboratory.

## Conclusions

Our results suggest that bone marrow suppression induced by administration of TS-1 in mice may be improved by coadministration of JTT. We used SELDI technology combined with SDS-PAGE and LC-MS/MS analysis to identify a specific albumin C-terminal fragment from plasma that transiently increases in mice treated with TS-1. Moreover, this fragment was decreased in the plasma of mice co-treated with JTT. The albumin fragment may be a suitable biomarker for predicting the onset of TS-1-induced myelosuppression. However, the sensitivity and specificity of the biomarker candidate must be validated in future clinical studies.

## Abbreviations

JTT: Juzentaihoto; WBC: White blood cell; BMC: Bone marrow cell; SELDI TOF-MS: Surface-enhanced laser desorption/ionization time-of-flight mass spectrometry.

## Competing interests

TY received a research grant from Tsumura & Co.; KO, CM, NT and MY are employees of Tsumura & Co.. Tsumura & Co. manufactures Juzentaihoto. The rest of authors declare that they have no competing interest.

## Authors’ contributions

K.O.: plasma collection, SELDI analysis, identification of biomarker candidate and draft the manuscript. T.O.: BMC collection and analysis. C.M.: plasma collection, SELDI analysis. N.T.: WBC analysis. M.Y.: direct and help with the experiments and draft of the manuscript. Y.N: conceive, plan and direct the research project. T.Y.: the study design. All authors read and approved the final manuscript.

## Pre-publication history

The pre-publication history for this paper can be accessed here:

http://www.biomedcentral.com/1472-6882/12/118/prepub
